# Insights into invasive fungal infection diagnostic and treatment capacities in tertiary care centres of Germany

**DOI:** 10.1093/jacamr/dlae083

**Published:** 2024-05-29

**Authors:** Jon Salmanton-García, Michaela Simon, Andreas H Groll, Oliver Kurzai, Tobias Lahmer, Thomas Lehrnbecher, Maria Schroeder, Oliver A Cornely, Jannik Stemler

**Affiliations:** Cologne Excellence Cluster on Cellular Stress Responses in Aging-Associated Diseases (CECAD), University of Cologne, University Hospital Cologne, Institute of Translational Research, Herderstraße 52, 50931 Cologne, Germany; Department I of Internal Medicine, Center for Integrated Oncology Aachen Bonn Cologne Duesseldorf (CIO ABCD) and Excellence Center for Medical Mycology (ECMM), University of Cologne, University Hospital Cologne, Cologne, Germany; German Centre for Infection Research (DZIF), Partner Site Bonn-Cologne, Cologne, Germany; Institute for Medical Microbiology, Immunology, and Hygiene, University Hospital Cologne and Faculty of Medicine, University of Cologne, Cologne, Germany; Infectious Disease Research Program, Center for Bone Marrow Transplantation and Department of Pediatric Hematology/Oncology, Children’s University Hospital Münster, Münster, Germany; National Reference Center for Invasive Fungal Infections, Leibniz Institute for Natural Product Research and Infection Biology, Hans Knoell Institute, Jena, Germany; TUM School of Medicine and Health, Department of Clinical Medicine—Clinical Department for Internal Medicine II, University Medical Centre, Technical University of Munich, Munich, Germany; Department of Pediatrics, Division of Hematology, Oncology and Hemostaseology, Goethe University Frankfurt, Frankfurt am Main, Germany; Department of Intensive Care Medicine, University Medical Center Hamburg-Eppendorf, Hamburg, Germany; Cologne Excellence Cluster on Cellular Stress Responses in Aging-Associated Diseases (CECAD), University of Cologne, University Hospital Cologne, Institute of Translational Research, Herderstraße 52, 50931 Cologne, Germany; Department I of Internal Medicine, Center for Integrated Oncology Aachen Bonn Cologne Duesseldorf (CIO ABCD) and Excellence Center for Medical Mycology (ECMM), University of Cologne, University Hospital Cologne, Cologne, Germany; German Centre for Infection Research (DZIF), Partner Site Bonn-Cologne, Cologne, Germany; Clinical Trials Centre Cologne (ZKS Köln), University of Cologne, University Hospital Cologne, Cologne, Germany; Cologne Excellence Cluster on Cellular Stress Responses in Aging-Associated Diseases (CECAD), University of Cologne, University Hospital Cologne, Institute of Translational Research, Herderstraße 52, 50931 Cologne, Germany; Department I of Internal Medicine, Center for Integrated Oncology Aachen Bonn Cologne Duesseldorf (CIO ABCD) and Excellence Center for Medical Mycology (ECMM), University of Cologne, University Hospital Cologne, Cologne, Germany; German Centre for Infection Research (DZIF), Partner Site Bonn-Cologne, Cologne, Germany

## Abstract

**Introduction:**

In Germany, the growing incidence of invasive fungal infections (IFIs) is a significant health concern, particularly impacting individuals with compromised immune systems due to factors like increasing transplant recipients, an ageing population, and heightened use of immunosuppressive medications. Diagnosing IFI remains challenging, and the integration of biomarker assays into clinical practice is difficult. Antifungal resistance, exemplified by pan-antifungal-resistant *Candida auris* cases, adds complexity to treatment. This study aims to provide a concise overview of the diagnostic and treatment landscape for IFI in Germany, identifying areas for improvement and paving the way for targeted interventions.

**Methods:**

Data were collected using an online electronic case report form from October 2021 to February 2023. The survey included questions about institutional practices related to fungal infection diagnosis and treatment, with invitations extended to researchers nationwide.

**Results:**

The study surveyed 58 hospitals across Germany. Notably, 77.6% managed high-risk patients for IFI. While 86% had onsite microbiology labs, a significant difference was noted for high-risk patients (93% in specialized hospitals versus 62% in others). Microscopy services had 96% coverage, while overall access to culture was 96%. Antigen tests had 96% coverage, and antibody access was reported at 98%. PCR testing was available at 98%. Imaging access showed no significant access differences. Variability existed in amphotericin B formulations based on patient profiles. Therapeutic drug monitoring was more common in high-risk patient institutions (89.5% versus 50.0%). All analysed institutions reported access to surgery (100%).

**Conclusions:**

Addressing identified disparities in diagnostic and therapeutic resources for IFI is crucial to improving patient outcomes. The study calls for ongoing research and collaboration to optimize strategies for the prevention and treatment of IFI, emphasizing the importance of equitable access to resources, especially in high-risk patient populations.

## Introduction

The surge in invasive fungal infections (IFIs) has emerged as a substantial health concern in Germany, especially impacting individuals with compromised immune systems. This increasing occurrence is linked to various factors, such as an increased number of individuals living with bone marrow or solid organ transplants (SOTs), ageing population, heightened use of immunosuppressive medications, and the alarming emergence of drug-resistant fungal pathogens.^1,2^

Diagnosing IFI is challenging and requires a large armamentarium of tools, some of which have been already described from the WHO as essential.^3,4^ While biomarker assays hold potential for providing faster and more precise diagnoses compared with traditional culture-based methods,^5,6^ their integration into clinical practice is still a challenge, mainly linked to its appropriate employment and proper interpretations.^5,7^ Moreover, the increasing issue of antifungal resistance, exemplified by numerous cases involving pan-antifungal-resistant *Candida auris*^8^ and other fungi with acquired resistances,^9,10^ adds complexity to identifying effective treatment strategies, posing a substantial threat to public health. Furthermore, the intricate and fragmented nature of the German healthcare system^11^ adds an additional layer of complexity in managing IFI. Access to specialized care, primarily concentrated in major metropolitan areas,^12^ is impeded by the decentralized structure, potentially making it challenging for patients to receive timely and appropriate treatment.

This study aims to furnish a comprehensive overview of the diagnostic and treatment landscape for IFI in Germany. In contrast to a previous and differently focused survey that was restricted to the setting of university-based medicine,^13^ this study provides a comprehensive overview of the availability of diagnostic and therapeutic resources for IFI in tertiary care hospitals across Germany. The majority of hospitals had access to mycology laboratories and were equipped to handle and treat IFI patients. However, significant disparities were described in the access to certain diagnostic and therapeutic resources, being more prevalent in the most specialized centres performing HSCT or SOT.

## Methods

Information was collected using an online electronic case report form (ECRF) hosted at www.clinicalsurveys.net/uc/IFI_management_capacity/ (EFS Summer 2021, TIVIAN GmbH, Cologne, Germany) between October 2021 and February 2023. Before analysis, the data were formally monitored to assure completeness and consistency. Queries were sent to gather information about (i) the institution, (ii) how often IFIs are diagnosed at the institution, (iii) microscopy, (iv) fungal cultures and identification, (v) serology, (vi) antigen detection, (vii) molecular assays, (viii) access to antifungal drugs and therapeutic drug monitoring (TDM) and (ix) access to imaging procedures like CT scan, MRI, ultrasound, X-ray and different endoscopies. Participants had to choose either ‘available’ or ‘not available’ for each technique at their institution. The overall perceived IFI incidence was rated on a Likert scale from 1 (very low) to 5 (very high) (Table S1, available as Supplementary data at *JAC-AMR* Online).

Scientists from all German federal states were invited to participate in the study. Invitations were sent out not only to the authors’ close collaborators but also to members of scientific organizations such as the International Society of Human and Animal Mycology (ISHAM), the European Confederation of Medical Mycology (ECMM) and the Infectious Diseases Working Group (AGIHO) of the German Society for Hematology and Medical Oncology (DGHO). Additionally, online calls for participation were posted on the LinkedIn^®^ and Twitter^®^ social media platforms.

Participating institutions were categorized based on the type of patients admitted, distinguishing between those performing HSCT or SOT and those not. The presentation of data involves frequencies and percentages, and proportions were organized in contingency tables. Statistical comparisons were made using Fisher’s exact test for variables with at least one cell with an expected value <5 and the chi-squared test for variables with all cells with expected values >5, as appropriate. A significance level of *P* < 0.05 was considered statistically significant. Statistical analyses were performed exclusively on valid responses in the respective summary or comparison. Consequently, centres with no responses were excluded from the analysis. Statistical analyses were conducted using SPSS v27.0 (SPSS, IBM Corp., Chicago, IL, USA).

## Results

**Figure 1.**
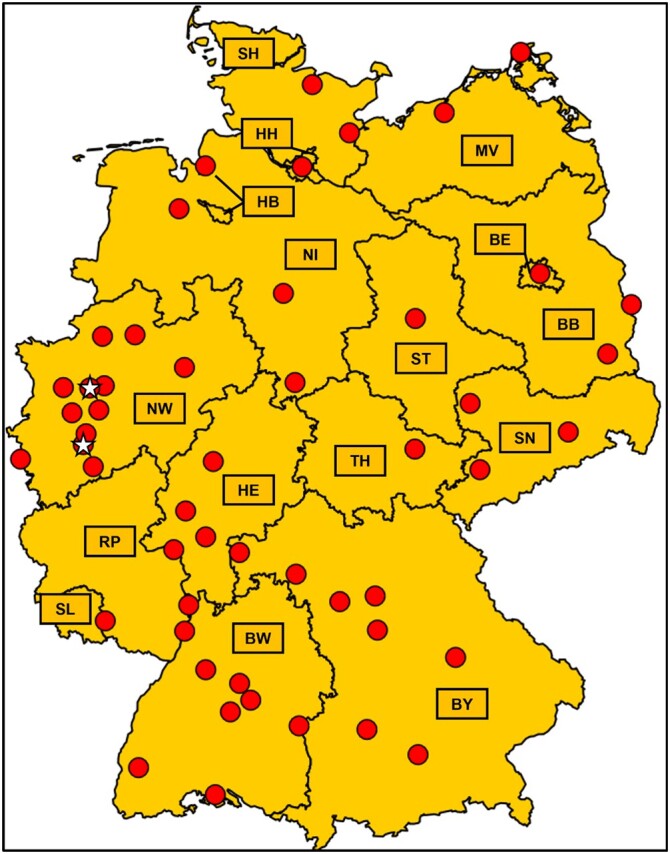
Map of participating institutions per country. White stars indicate the University Hospital Cologne and University Hospital Essen, respectively. These institutions are granted ECMM Diamond Excellence Center Status.^14^ BB, Brandenburg (2 centres); BE, Berlin (2 centres); BW, Baden-Württemberg (9 centres); BY, Bavaria (10 centres); HB, Bremen (1 centre); HH, Hamburg (1 centre); HE, Hesse (3 centres); NI, Lower Saxony (3 centres); MV, Mecklenburg-Vorpommern (2 centres); NW, North Rhine-Westphalia (16 centres); RP, Rhineland-Palatinate (1 centre); SL, Saarland (1 centre); SN, Saxony (3 centres); ST, Saxony-Anhalt (1 centre); SH, Schleswig-Holstein (2 centres); TH, Thuringia (1 centre).

Data were gathered from 58 hospitals throughout Germany, with at least one hospital from each of the 16 German Federal States (Figure 1). The analysis revealed that 43 out of the 58 hospitals (74.1%) were equipped to handle SOTs, and 42 out of 58 (72.4%) were capable of conducting HSCT. In total, 45 out of 58 (77.6%) hospitals were managing such patients at high risk for IFI (Table 1).

**Table 1.** Baseline characteristics of participating institutions in Germany

Characteristic	Total		HSCT/SOT		No HSCT/SOT		*P* value
	*n*/*N*	%	*n*/*N*	%	*n*/*N*	%	
Type of patients							
COVID-19	58/58	100.0	45/45	100.0	13/13	100.0	1.000
Diabetes mellitus	58/58	100.0	45/45	100.0	13/13	100.0	1.000
Haematology	57/58	98.3	44/45	97.8	13/13	100.0	1.000
HIV/AIDS	50/58	86.2	45/45	100.0	5/13	38.5	<0.001*
Adult ICU	36/38	94.7	29/31	93.5	7/7	100.0	1.000
Neonatal ICU	51/58	87.9	43/45	95.6	8/13	61.5	0.005*
Oncology	58/58	100.0	45/45	100.0	13/13	100.0	1.000
Total parenteral nutrition	57/58	98.3	44/45	97.8	13/13	100.0	1.000
Respiratory/other intermediate ICU	37/38	97.4	31/31	100.0	6/7	85.7	0.184
SOT	43/58	74.1	43/45	95.6	0/13	0.0	<0.001*
Stem cell transplantation	42/58	72.4	42/45	93.3	0/13	0.0	<0.001*
Microbiology laboratory							
Onsite	50/58	86.2	42/45	93.3	8/13	61.5	0.010*
Outsourced	8/58	13.8	3/45	6.7	5/13	38.5	
Mycological diagnosis performance							
Onsite	35/58	60.3	30/45	66.7	5/13	38.5	0.002*
Onsite—outsourced	19/58	32.8	15/45	33.3	4/13	30.8	
Outsourced	4/58	6.9	0/45	0.0	4/13	30.8	
Mycologist							
Onsite	23/38	60.5	23/31	74.2	0/7	0.0	<0.001*
Onsite—outsourced	6/38	15.8	5/31	16.1	1/7	14.3	
Outsourced	3/38	7.9	0/31	0.0	3/7	42.9	
No access	6/38	15.8	3/31	9.7	3/7	42.9	
Clinician notification system							
Laboratory information system	26/29	89.7	22/24	91.7	4/5	80.0	0.446
Pager/beeper	4/26	15.4	4/22	18.2	0/4	0.0	1.000
Telephone call	31/32	96.9	27/27	100.0	4/5	80.0	0.156
Text message	1/27	3.7	1/23	4.3	0/4	0.0	1.000
Consultation to reference laboratories	23/33	69.7	21/27	77.8	2/6	33.3	0.053
IFI incidence perception							
Very low	5/52	9.6	3/42	7.1	2/10	20.0	0.692
Low	27/52	51.9	21/42	50.0	6/10	60.0	
Moderate	16/52	30.8	14/42	33.3	2/10	20.0	
High	2/52	3.8	2/42	4.8	0/10	0.0	
Very high	2/52	3.8	2/42	4.8	0/10	0.0	
Most important pathogen(s)							
*Aspergillus* spp.	49/53	92.5	42/43	97.7	7/10	70.0	0.018*
*Candida* spp.	49/53	92.5	40/43	93.0	9/10	90.0	1.000
*Cryptococcus* spp.	11/53	20.8	11/43	25.6	0/10	0.0	0.098
*Fusarium* spp.	5/53	9.4	5/43	11.6	0/10	0.0	0.570
*Histoplasma* spp.	1/53	1.9	1/43	2.3	0/10	0.0	1.000
*Lomentospora/Scedosporium* spp.	1/34	2.9	1/29	3.4	0/5	0.0	1.000
Mucorales	14/53	26.4	14/43	32.6	0/10	0.0	0.047
Phaeohyphomycetes	1/34	2.9	1/29	3.4	0/5	0.0	1.000

^*^indicates statistically significant difference.

The overall availability of onsite access to a microbiology laboratory was widespread (50 out of 58 hospitals; 86.2%). However, a significant disparity in onsite laboratory access was observed when examining patients at the highest risk for IFI, specifically those undergoing HSCT or SOT. In 42 out of 45 hospitals (93.3%) capable of treating such high-risk patients, onsite laboratories were available, in contrast to 8 out of 13 hospitals (61.5%) without this specialized treatment portfolio (*P* = 0.010). Similarly, a significant difference was noted in onsite access to at least one mycological diagnostic test, with 45 out of 45 hospitals (100%) with high-risk patients having such access compared with 9 out of 13 hospitals (69.2%; *P* = 0.002) without. However, no statistically significant difference was identified in the communication methods for clinical results between laboratory and clinical mycologists, with telephone calls (31 out of 32 hospitals; 96.9%) and laboratory information systems (26 out of 29 hospitals; 89.7%) being the most prevalent approaches (Table 1).

Participants were asked about their experiential insights to estimate the incidence of IFI within their respective institutions. Responses predominantly fell into the categories of moderate (16 out of 52; 30.8%) and low (27 out of 52; 51.9%). Specifically, when asked to identify pathogens of concern based on their local IFI epidemiology, *Aspergillus* spp. and *Candida* spp. emerged as the most commonly reported, with both pathogens selected by 49 out of 53 participants (92.5%). Notably, *Aspergillus* spp. were perceived as more concerning in institutions with HSCT/SOT patients compared with those without (97.7% versus 70.0%; *P* = 0.018) (Table 1).

Regarding microbiological techniques, microscopy was available in 49 out of 51 respondent institutions, representing 96.1% coverage. Institutions handling HSCT/SOT patients exhibited a higher rate of microscopy services, with 41 out of 42 (97.6%), compared with 8 out of 9 (88.9%) in institutions without such patients. Significant differences were observed in access to fluorescence dye (83.3% versus 44.4%; *P* = 0.024) (Table 1).

The overall access to culture was higher in institutions with HSCT/SOT patients (97.6% versus 87.5%), although the same rates were observed for species identification tests (100.0% each), and similarly for antifungal susceptibility testing (97.5% versus 87.5%). No differences were observed when comparing access to antifungal susceptibility testing of moulds and/or yeasts between centres. Moreover, no significant differences were noted in access to antibody (97.5% versus 100.0%) and antigen detection (97.4% versus 85.7%), as well as molecular tests (100.0% versus 85.7%). Of note, such novel diagnostic approaches were more frequently observed onsite rather than outsourced in centres managing HSCT and SOT patients (Table 2).

**Table 2.** Access to laboratory tools of participating institutions in Germany

Laboratory tool	Total		HSCT/SOT		No HSCT/SOT		*P* value
	*n*/*N*	%	*n*/*N*	%	*n*/*N*	%	
Microscopy	49/51	96.1	41/42	97.6	8/9	88.9	0.325
Methodologies							
Calcofluor white	30/39	76.9	26/35	74.3	4/4	100.0	0.556
Giemsa stain	33/43	76.7	28/38	73.7	5/5	100.0	0.320
China/India ink	34/43	79.1	30/38	78.9	4/5	80.0	1.000
Potassium hydroxide	18/35	51.4	17/33	51.5	1/2	50.0	1.000
Silver stain	30/48	62.5	23/40	57.5	7/8	87.5	0.229
Access to fluorescence	39/51	76.5	35/42	83.3	4/9	44.4	0.024*
Direct examination if cryptococcosis suspected	46/51	90.2	40/42	95.2	6/9	66.7	0.033*
Silver stain if pneumocystis suspected	24/51	47.1	18/42	42.9	6/9	66.7	0.276
Direct microscopy if mucormycosis suspected	33/51	64.7	29/42	69.0	4/9	44.4	0.249
Culture	47/49	95.9	40/41	97.6	7/8	87.5	0.303
Fungal culture methods							
Agar Niger	10/32	31.3	10/31	32.3	0/1	0.0	1.000
Chromogen	26/29	89.7	26/28	92.9	0/1	0.0	0.103
Lactrimel agar	3/32	9.4	3/31	9.7	0/1	0.0	1.000
Potato dextrose agar	19/32	59.4	19/31	61.3	0/1	0.0	0.406
Sabouraud dextrose agar	30/35	85.7	29/34	85.3	1/1	100.0	1.000
Sabouraud dextrose agar + chloramphenicol	23/32	71.9	23/31	74.2	0/1	0.0	0.281
Sabouraud dextrose agar + gentamicin	18/32	56.3	18/31	58.1	0/1	0.0	0.437
Selective agar	16/32	50.0	16/31	51.6	0/1	0.0	1.000
Available tests for specific identification	47/47	100.0	40/40	100.0	7/7	100.0	1.000
Automated identification	37/45	82.2	33/40	82.5	4/5	80.0	1.000
Biochemical tests	30/43	69.8	27/39	69.2	3/4	75.0	1.000
DNA sequencing	33/43	76.7	33/39	84.6	0/4	0.0	0.002*
MALDI—TOF MS	39/43	90.7	35/38	92.1	4/5	80.0	0.402
Mounting medium	21/35	60.0	19/32	59.4	2/3	66.7	1.000
Antifungal susceptibility testing	46/48	95.8	39/40	97.5	7/8	87.5	0.309
For yeasts	13/46	28.3	10/39	25.6	3/7	42.9	
For both	33/46	71.7	29/39	74.4	4/7	57.1	
Antifungal susceptibility methodology							
CLSI	8/36	22.2	8/34	23.5	0/2	0.0	1.000
EUCAST	29/40	72.5	27/38	71.1	2/2	100.0	1.000
Gradient strip test	31/38	81.6	30/35	85.7	1/3	33.3	0.081
Semiautomated antifungal susceptibility testing system	26/38	68.4	24/35	68.6	2/3	66.7	1.000
Antibody detection	45/46	97.8	39/40	97.5	6/6	100.0	1.000
*Aspergillus* spp.	44/45	97.8	38/39	97.4	6/6	100.0	1.000
Onsite	35/44	79.5	31/38	81.6	4/6	66.7	0.586
Outsourced	9/44	20.5	7/38	18.4	2/6	33.3	
*Candida* spp.	38/45	84.4	34/40	85.0	4/5	80.0	1.000
Onsite	27/38	71.1	24/34	70.6	3/4	75.0	1.000
Outsourced	11/38	28.9	10/34	29.4	1/4	25.0	
*Histoplasma* spp.	34/41	82.9	29/35	82.9	5/6	83.3	1.000
Onsite	7/34	20.6	5/29	17.2	2/5	40.0	0.268
Outsourced	27/34	79.4	24/29	82.8	3/5	60.0	
Antigen detection	44/46	95.7	38/39	97.4	6/7	85.7	0.284
*Aspergillus* galactomannan, any	43/44	97.7	38/38	100.0	5/6	83.3	0.136
*Aspergillus* galactomannan (ELISA)	41/43	95.3	36/37	97.3	5/6	83.3	0.262
Onsite	37/41	90.2	33/36	91.7	4/5	80.0	0.418
Outsourced	4/41	9.8	3/36	8.3	1/5	20.0	
*Aspergillus* galactomannan (LFA)	14/40	35.0	12/35	34.3	2/5	40.0	1.000
Onsite	10/14	71.4	9/12	75.0	1/2	50.0	0.505
Outsourced	4/14	28.6	3/12	25.0	1/2	50.0	
*Aspergillus* galactomannan (LFD)	10/38	26.3	8/35	22.9	2/3	66.7	0.164
Onsite	6/10	60.0	5/8	62.5	1/2	50.0	1.000
Outsourced	4/10	40.0	3/8	37.5	1/2	50.0	
*Candida* antigen	36/43	83.7	32/38	84.2	4/5	80.0	1.000
Onsite	26/36	72.2	23/32	71.9	3/4	75.0	1.000
Outsourced	10/36	27.8	9/32	28.1	1/4	25.0	
*Cryptococcus* mannan, any	42/43	97.7	37/37	100.0	5/6	83.3	0.140
*Cryptococcus* (LAT)	34/43	79.1	30/37	81.1	4/6	66.7	0.589
Onsite	24/34	70.6	22/30	73.3	2/4	50.0	0.564
Outsourced	10/34	29.4	8/30	26.7	2/4	50.0	
*Cryptococcus* (LFA)	24/39	61.5	20/34	58.8	4/5	80.0	0.631
Onsite	17/24	70.8	15/20	75.0	2/4	50.0	0.552
Outsourced	7/24	29.2	5/20	25.0	2/4	50.0	
*Histoplasma* antigen	28/40	70.0	24/34	70.6	4/6	66.7	1.000
Onsite	6/28	21.4	5/24	20.8	1/4	25.0	1.000
Outsourced	22/28	78.6	19/24	79.2	3/4	75.0	
β-D-glucan	27/41	65.9	23/34	67.6	4/7	57.1	0.673
Onsite	13/27	48.1	10/23	43.5	3/4	75.0	0.326
Outsourced	14/27	51.9	13/23	56.5	1/4	25.0	
Molecular tests	44/45	97.8	38/38	100.0	6/7	85.7	0.156
*Aspergillus* PCR	35/43	81.4	31/37	83.8	4/6	66.7	0.308
Onsite	25/35	71.4	24/31	77.4	1/4	25.0	0.061
Outsourced	10/35	28.6	7/31	22.6	3/4	75.0	
*Candida* PCR	29/40	72.5	26/35	74.3	3/5	60.0	0.603
Onsite	18/29	62.1	18/26	69.2	0/3	0.0	0.045*
Outsourced	11/29	37.9	8/26	30.8	3/3	100.0	
*Pneumocystis* PCR	43/44	97.7	37/37	100.0	6/7	85.7	0.159
Onsite	39/43	90.7	35/37	94.6	4/6	66.7	0.087
Outsourced	4/43	9.3	2/37	5.4	2/6	33.3	
Mucorales PCR	28/41	68.3	24/35	68.6	4/6	66.7	1.000
Onsite	13/28	46.4	13/24	54.2	0/4	0.0	0.102
Outsourced	15/28	53.6	11/24	45.8	4/4	100.0	
TDM	38/46	82.6	34/38	89.5	4/8	50.0	0.022*
Flucytosine	17/40	42.5	15/33	45.5	2/7	28.6	0.677
Isavuconazole	13/23	56.5	11/20	55.0	2/3	66.7	1.000
Itraconazole	23/39	59.0	21/32	65.6	2/7	28.6	0.085
Posaconazole	33/40	82.5	30/34	88.2	3/6	50.0	0.055
Voriconazole	38/43	88.4	34/36	94.4	4/7	57.1	0.024*

^*^indicates statistically significant difference.

LAT, latex agglutination test; LFA, lateral flow assay; LFD, lateral flow device.

**Figure 2.**
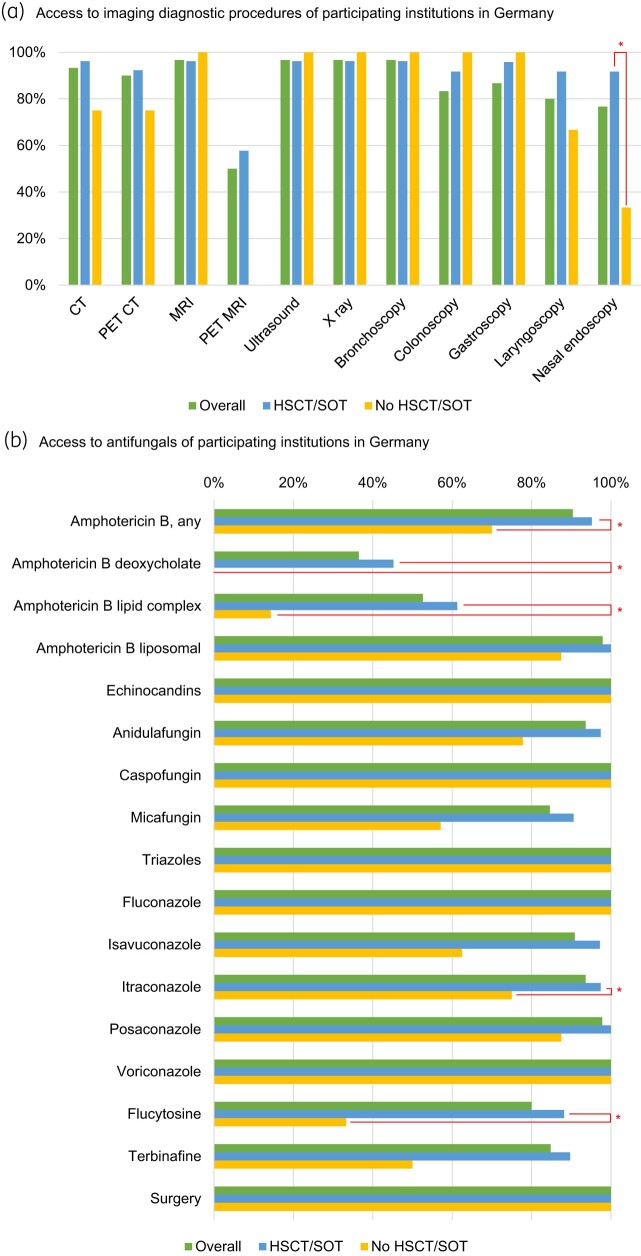
Access to imaging diagnostic and treatment tools of participating institutions in Germany. (a) Access to imaging diagnostic procedures of participating institutions in Germany. Asterisks (*) indicate statistically significant difference. Full details and comparisons are presented in Table S1. (b) Access to antifungals of participating institutions in Germany. Asterisks (*) indicate statistically significant difference. Full details and comparisons are presented in Table S1.

No significant differences were observed between centres with HSCT/SOT patients and those without in terms of access to X-rays, ultrasound, CT, positron emission tomography (PET) CT, MRI, PET MRI or endoscopy. The only notable distinction was found in the access to nasal endoscopy, where 22 out of 24 institutions with HSCT/SOT patients (91.7%) had access, compared with only 1 out of 3 institutions without such patients (33.3%; *P* = 0.049) (Figure 2a, Table S1).

Overall access to at least one formulation of amphotericin B was reported in 90.4% (47/52) of the surveyed institutions. Statistically significant differences were observed in the general access to any amphotericin B formulation (*P* = 0.043), as well as to two of the formulations: deoxycholate (*P* = 0.006) and lipid complex (*P* = 0.038). Access to all these formulations was more prevalent in centres handling HSCT/SOT patients. Lipid complex (14.3%) and liposomal (87.5%) were the only amphotericin B formulations available in centres without HSCT/SOT patients. For echinocandins, overall access reached 100.0% (49/49), with no significant differences between centres. However, access was higher in HSCT/SOT centres, particularly for micafungin (90.6% versus 57.1%). Access to at least one triazole was homogeneous (100.0% each group). Yet, for mould-active triazoles, significant differences were observed in access to itraconazole (97.4% versus 75.0%; *P* = 0.015) (Figure 2b, Table S1).

TDM was more common in institutions treating high-risk patients (89.5% versus 50.0%; *P* = 0.022), particularly for voriconazole (94.4% versus 57.1%; *P* = 0.024). Access to surgery was reported by all analysed institutions (*n* = 27).

## Discussion

This survey of 58 hospitals across Germany sheds light on capabilities and disparities in the management of IFI patients. The study provides a comprehensive overview of the availability of diagnostic and therapeutic resources for IFI in tertiary care hospitals across Germany. The majority of hospitals had access to mycology laboratories and were equipped to handle and treat IFI patients. However, significant disparities were described in the access to certain diagnostic and therapeutic resources, being more prevalent in the most specialized centres performing HSCT or SOT.

Participating researchers’ feedback into the incidence of IFI within their institutions provides valuable qualitative data. The majority of responses indicate a moderate or low incidence, possibly attributed to (i) the lack of endemic pathogenic fungi in Germany,^15^ (ii) the successful local management of underlying conditions such as diabetes mellitus or HIV/AIDS^1,16–18^ or (iii) the effective implementation of prophylactic approaches in immunosuppressed patients.^19^ The identification of *Aspergillus* spp. and *Candida* spp. as the most reported pathogens aligns with existing literature from neighbouring countries and on a continental scale, as with the fungal priority pathogens list of the WHO.^20–23^ It is noteworthy that there is a perception that *Aspergillus* spp. are of greater concern in institutions with HSCT/SOT patients,^24–27^ aligning with the epidemiology of IFIs and the distribution of pathogens in HSCT patients, despite not that much in SOT.^28^

**Figure 3.**
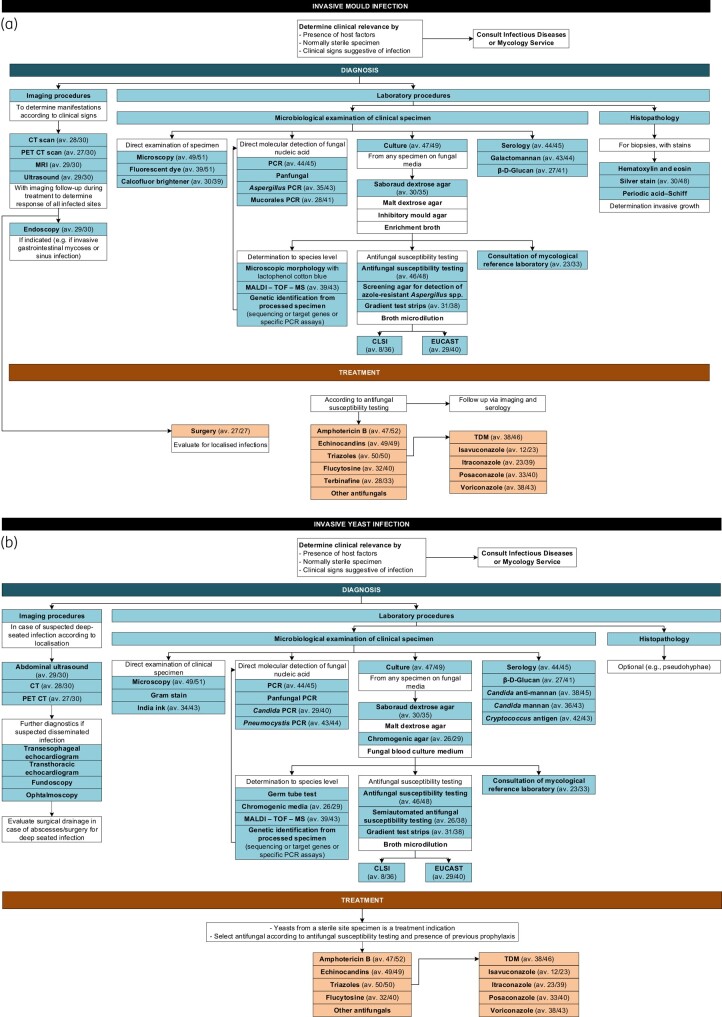
Guideline-recommended IFI management flowchart including the access to diagnostic and treatment tools of participating institutions in Germany. (a) Invasive mould infection management. (b) Invasive yeast infection management. av., available (in xx/yy sites).

The higher prevalence of onsite microscopy services and access to specialized microscopy procedures in HSCT/SOT centres support that these institutions are better prepared to diagnose IFI promptly. This is particularly important in the setting of HSCT and SOT, where patients are at increased risk of IFI due to immunosuppression.^15,29–33^ The ability to perform rapid microscopy can aid in the early identification of fungal pathogens, allowing for the timely initiation of appropriate antifungal therapy. Similarly, the greater availability of culture methods, antibody detection, antigen detection and molecular tests in HSCT/SOT centres highlights their enhanced capacity for comprehensive diagnostic evaluation. These methods provide valuable information for confirming the diagnosis of IFI, in line with the recommendations of the WHO,^4^ identifying the specific fungal pathogen involved, and determining antifungal susceptibility patterns. This information is crucial for guiding the selection of the most effective antifungal therapy,^15,30–33^ particularly given the growing challenge of resistance in the currently available arsenal against specific pathogenic species. Nevertheless, while most of the analysed sites manage to adhere to guideline recommendations, there are still some that fail to offer all the necessary elements for a comprehensive patient journey. Consequently, a federal initiative is essential to ensure effective IFI management, irrespective of the patient’s location within our country (Figure 3).

The observed disparities in the accessibility of antifungal agents, specifically encompassing amphotericin B formulations, mould-active triazoles, flucytosine and terbinafine, between centres that specialized in HSCT/SOT and those that did not, underscore the imperative for meticulous refinement of antifungal drug formularies within broader healthcare institutions catering to non-high-risk patient populations, in accordance with the WHO essential medicines list.^3^ By rectifying these discrepancies and instituting strategies to augment the accessibility of essential antifungal agents, healthcare institutions can enhance their capacity to handle IFI across a wider range of patients, in line with current guidelines.^15,30–34^ This holds particular significance given the rising incidence of IFI among immunocompetent individuals, attributable to factors such as travel history to endemic regions,^35,36^ burns^37^ or traumatic injuries,^38^ and more recently as a secondary infection to respiratory viral infections, including influenza- or severe acute respiratory syndrome coronavirus type 2 (SARS-CoV-2)-associated pulmonary aspergillosis.^9,39^

The widespread availability of TDM, especially for posaconazole and voriconazole, in HSCT/SOT centres demonstrates their commitment to optimizing antifungal therapy. TDM allows for the measurement of antifungal drug concentrations in the patient’s blood, ensuring that therapeutic levels are maintained and minimizing the risk of toxicity.^15,30–33^ This is particularly important for voriconazole, which has a narrow therapeutic window.

The study has several limitations. First, its cross-sectional design impedes the inference of causal relationships. Additionally, reliance on self-reported data introduces potential biases. A prospective study with standardized data collection methods would offer stronger evidence and diminish the risk of bias. Moreover, the data collection period spans from October 2021 to February 2023, potentially limiting the generalizability of the findings to this timeframe. Furthermore, the study’s focus on tertiary care hospitals may not fully represent the broader healthcare landscape in Germany, with many secondary care hospitals. In parallel, it is conceivable that tertiary care centres, renowned for their specialized expertise and resources in managing complex medical conditions, along with members of mycology societies, who likely possess a heightened interest and proficiency in treating IFI, may have been disproportionately represented within the study cohort. These entities are often at the forefront of IFI diagnosis, treatment and research, potentially leading to their overrepresentation in the study sample due to their inherent inclination and capacity to contribute to such research endeavours. Future analyses should incentivize a more transversal participation of German healthcare providers. In a few years, it would be opportune to thoroughly explore this constraint, given the ongoing transformation of the hospital scenario resulting from the legal reforms in Germany. Despite these limitations, the study provides first insights into the diagnostic and treatment landscape for IFI in Germany, offering a foundation for targeted interventions and improvements in IFI management.

In conclusion, this study highlights the importance of ensuring equitable access to diagnostic and therapeutic resources for IFI, particularly in high-risk patient populations. By identifying disparities, institutions can improve the management of IFI and enhance patient outcomes. Additionally, ongoing research and collaboration among healthcare providers, policymakers and pharmaceutical companies are crucial to optimize antifungal prophylaxis, empirical therapy and TDM strategies for the prevention and treatment of IFI.

## Supplementary Material

dlae083_Supplementary_Data

## Collaborators

Peter Abel, Alexander Halfmann, Alexander Link, Andreas Zautner, Anja Berger, Anna Dudakova, Anna Goer, Annika Schmidt, Armin Hoffmann, Antonia Sassmann-Schweda, Axel Hamprecht, Beniam Ghebremedhin, Benigno Florido Prados, Bernhard Zöllner, Christian Haas, Christina Bahrs, Claudia Crowell, Karl Dichtl, Elena Busch, Elham Khatamzas, Enrico Schalk, Felix Goeser, Helmut Fickenscher, Johannes Forster, Franziska Ebeling, Frieder Fuchs, Frieder Schaumburg, Fuat Saner, Gerhard Haase, Gregor Paul, Lior Haftel, Janina Hammer, Hans Martin Orth, Helmut Ostermann, Helmut Schweiger, Herbert Hof, Claus Peter Heußel, Ioana Diana Olaru, Janina Trauth, Jochen Brasch, Joerg Steinmann, Jürgen Bohnert, Jürgen Held, Jürgen Rödel, Juliane Brandt, Karsten Becker, Kathrin Oldenburg, Kerstin Arndt, Khalid Shoumariyeh, Martina Kerwat, Michael Krakau, Leander Köpnick, Lukas Eberwein, Lutz von Müller, Maximilian Desole, Marlis Gerigk, Martha Oberschelp, Martina Furitsch, Martina Teichmann, Matthias Vöhringer, Meinolf Karthaus, Melanie Eisele, Micha Lobermann, Michael Kiehl, Michael Schmid, Modar Saleh, Mohamed Karam Douedari, Ernst Molitor, Marc Stadler, Nadine Dietze, Nael Alakel, Norman Lippmann, Philipp Koehler, Peter Rath, Susanne Elsner, Sabrina Klein, Sandra Gräber, Oliver Schildgen, Britt Schwenz, Ludwig Sedlacek, Soeren Becker, Jan Springer, Stefan Büttner, Stefan Glöckner, Stefan Schwartz, Stefanie Sammet, Susann Rößler, Susanne Hauswaldt, Tatjana Buchberger, Thomas Harrer, Timo Schmitt, Christoph Wyen, Sabine Ehrlich, Sebastian Grund, Walter Geißdörfer, Wolfgang Rabsch.

## Supplementary data

Table S1 is available as Supplementary data at *JAC-AMR* Online.
