# Supplementary material for: Insights into invasive fungal infection diagnostic and treatment capacities in tertiary care centres of Germany
Source: JAC Antimicrob Resist. 2024 May 29;6(3):dlae083. doi: 10.1093/jacamr/dlae083 (PMC11135635; doi:10.1093/jacamr/dlae083)
Supplement: dlae083_Supplementary_Data [file dlae083_supplementary_data.docx]

**Supplementary tables**

**Supplementary table 1.** Access to imaging diagnostic and treatment tools of participating institutions in Germany.

|  | **Total** | | **HSCT/SOT** | | **No HSCT/SOT** | | **p value** |
| --- | --- | --- | --- | --- | --- | --- | --- |
|  | ***n*** | ***%*** | ***n*** | ***%*** | ***n*** | ***%*** |  |
| **Imaging procedures** |  |  |  |  |  |  |  |
| CT | 28/30 | 93.3 | 25/26 | 96.2 | 3/4 | 75.0 | 0.253 |
| PET CT | 27/30 | 90.0 | 24/26 | 92.3 | 3/4 | 75.0 | 0.360 |
| MRI | 29/30 | 96.7 | 25/26 | 96.2 | 4/4 | 100.0 | 1.000 |
| Ultrasound | 29/30 | 96.7 | 25/26 | 96.2 | 4/4 | 100.0 | 1.000 |
| X ray | 29/30 | 96.7 | 25/26 | 96.2 | 4/4 | 100.0 | 1.000 |
| Bronchoscopy | 29/30 | 96.7 | 25/26 | 96.2 | 4/4 | 100.0 | 1.000 |
| Colonoscopy | 25/27 | 83.3 | 22/24 | 91.7 | 3/3 | 100.0 | 1.000 |
| Gastroscopy | 26/27 | 86.7 | 23/24 | 95.8 | 3/3 | 100.0 | 1.000 |
| Laryngoscopy | 24/27 | 80.0 | 22/24 | 91.7 | 2/3 | 66.7 | 0.308 |
| Nasal endoscopy | 23/27 | 76.7 | 22/24 | 91.7 | 1/3 | 33.3 | 0.049 * |
| **Available antifungals** |  |  |  |  |  |  |  |
| Amphotericin B | 47/52 | 90.4 | 40/42 | 95.2 | 7/10 | 70.0 | 0.043 * |
| *Amphotericin B deoxycholate* | 19/52 | 36.5 | 19/42 | 45.2 | 0/10 | 0.0 | 0.006 * |
| *Amphotericin B lipid complex* | 20/38 | 52.6 | 19/31 | 61.3 | 1/7 | 14.3 | 0.038 * |
| *Amphotericin B liposomal* | 47/48 | 97.9 | 40/40 | 100.0 | 7/8 | 87.5 | 0.167 |
| Echinocandins | 49/49 | 100.0 | 39/39 | 100.0 | 10/10 | 100.0 | 1.000 |
| *Anidulafungin* | 44/47 | 93.6 | 37/38 | 97.4 | 7/9 | 77.8 | 0.090 |
| *Caspofungin* | 49/49 | 100.0 | 39/39 | 100.0 | 10/10 | 100.0 | 1.000 |
| *Micafungin* | 33/39 | 84.6 | 29/32 | 90.6 | 4/7 | 57.1 | 0.059 |
| Triazoles | 50/50 | 100.0 | 40/40 | 100.0 | 10/10 | 100.0 | 1.000 |
| *Fluconazole* | 50/50 | 100.0 | 40/40 | 100.0 | 10/10 | 100.0 | 1.000 |
| *Isavuconazole* | 40/44 | 90.9 | 35/36 | 97.2 | 5/8 | 62.5 | 0.071 |
| *Itraconazole* | 44/47 | 93.6 | 38/39 | 97.4 | 6/8 | 75.0 | 0.015 * |
| *Posaconazole* | 45/46 | 97.8 | 38/38 | 100.0 | 7/8 | 87.5 | 0.174 |
| *Voriconazole* | 49/49 | 100.0 | 40/40 | 100.0 | 9/9 | 100.0 | 1.000 |
| Flucytosine | 32/40 | 80.0 | 30/34 | 88.2 | 2/6 | 33.3 | 0.010 * |
| Terbinafine | 28/33 | 84.8 | 26/29 | 89.7 | 2/4 | 50.0 | 0.099 |
| **Surgery** | 27/27 | 100.0 | 23/23 | 100.0 | 4/4 | 100.0 | 1.000 |

* statistically significant difference

**CT**, computed tomography; **HSCT/SOT**, hematopoietic stem cell transplantation/solid organ transplantation; **MRI**, magnetic resonance imaging; **PET**, positron emission tomography
